# Clinical epidemiology of easily recognisable congenital malformations in Gambian newborns: a descriptive cohort study embedded in a clinical trial

**DOI:** 10.1186/s12884-026-09673-2

**Published:** 2026-09-14

**Authors:** Shashu Graves, Nathalie Beloum, Bully Camara, Usman N. Nakakana, Madikoi Danso, Fatoumata Sillah, Joquina C. Jones, Edrissa Sabally, Siaka Badjie, Omar Jarra, Sulayman Bah, Abdoulie Suso, Ebrahim Ndure, Yusupha Njie, Christian Bottomley, Halidou Tinto, Umberto D’Alessandro, Helen Brotherton, Anna Roca

**Affiliations:** 1 The Elgin Clinic (Inclusive Health Primary Care Network - NHS), London, England; 2 Medical Research Council Unit, The Gambia at London School of Hygiene and Tropical Medicine (MRCG at LSHTM), Banjul, The Gambia; 3 Gates Foundation, Seattle, USA; 4 London School of Hygiene and Tropical Medicine, London, UK; 5 Institut de Recherche en Sciences de la Sante-Clinical Research Unit of Nanoro (IRSS-CRUN), Nanoro, Burkina Faso; 6 Victoria Hospital Kirkcaldy, NHS Fife, Kirkcaldy, UK; 7 ICREA Research Professor, Pg. Lluís Companys 23, Barcelona, 08010 Spain; 8 ISGlobal, Barcelona Global Health Institute, Barcelona, 08036 Spain

**Keywords:** Congenital Malformation, Neonates, Risk Factors, Mortality, Intrapartum-related asphyxia, West Africa

## Abstract

**Background:**

The prevalence of congenital malformations remains high and significantly contributes to neonatal morbidity and mortality in West Africa. However, there is a gap in the existing literature regarding the clinical epidemiology of congenital malformations in the African subregion. This study aimed to describe the clinical presentation, prevalence, risk factors, and adverse neonatal outcomes of newborns with easily recognisable congenital malformations in urban Gambia.

**Methods:**

This descriptive cohort study consisted of secondary analyses of a clinical trial data (PregnAnZi-2 trial) for liveborn neonates delivered between October 2017 to May 2021 at two public health facilities in The Gambia. Congenital malformations were detected by clinical examination at birth with active and passive surveillance for 28 days after delivery. Congenital malformations were classified according to ICD11 definitions, with sub-classification into major or minor malformations as per WHO guidance.

**Results:**

One hundred forty out of 6750 neonates (2.1%) had at least one congenital malformation, with 29.3% (44/150) of these malformations classified as major. The musculoskeletal system was most frequently affected (58%, 87/150) with talipes equinovarus as the most common major malformation identified (43%, 19/44). A history of previous miscarriage was associated with an increased risk of congenital malformations (OR:1.72, 95%CI:1.02–2.73, *p*-value = 0.04). Newborns with a congenital malformation were more likely to experience adverse neonatal outcomes than newborns without, with higher odds of low 1-min Apgar score, (OR:3.09, 95%CI:1.62–5.48, *p*-value < 0.001), low 5-min Apgar score (OR:6.10, 95%CI:2.10–14.62, *p*-value < 0.001), hospitalisation during the neonatal period (OR:4.42, 95%CI:2.77–6.83, *p*-value < 0.001) and mortality within 24h of delivery (OR 48.75, 95%CI:11.08–215.62, *p*-value < 0.001). Among 70 neonatal deaths recorded by day 28 after birth in the study, 15 (21.4%) were newborns with congenital malformation. The circulatory and skeletal systems were equally more affected among congenital malformations recorded on neonatal deaths by day 28 after birth (23.8%, 5/21).

**Conclusion:**

Despite a relatively low prevalence, easily recognisable congenital malformations are associated with substantial perinatal morbidity and mortality. Improved antenatal diagnosis of congenital malformations is urgently required to optimise delivery strategies and improve perinatal outcomes, especially for women experiencing previous miscarriage.

**Trial registration:**

NCT03199547: Clinicaltrials.gov. Registered on 23rd June 2017

DOI: https://dx.doi.org/10.18203/2349-3259.ijct20220110

## Introduction

Globally, congenital malformations affect approximately 6% of newborns, representing more than 8 million babies annually [1, 2]. Among these cases, major malformations, which involve a structural change with significant medical, social, or cosmetic consequences or require medical or surgical intervention [3], occur in 2–3% of live births and contribute to 20–30% of stillbirths [4]. However, there is a significant geographical variation in the prevalence of congenital malformations, with 94% of newborns with a major congenital malformation in low- and middle-income countries [1, 5].

As global neonatal mortality rates decline, the proportion of neonatal deaths attributed to congenital malformations has increased [6]. Congenital malformations account for 6% of neonatal deaths [6], with 95% of these deaths occurring in low- and middle-income countries [1, 5]. For those surviving the neonatal period, congenital malformations can lead to life-long consequences, including chronic illness, long-term disability, and premature death. They account for 25 million disability-adjusted life years (DALYs) globally and have a substantial impact on lost life years (LLYs) [1, 7–10].

Congenital malformations can be classified according to the underlying developmental mechanism. About one-third of congenital malformations might have a genetic cause, with 17% related to single-gene abnormalities and 10% to chromosomal changes [11]. Another ~ 10% are associated with environmental and maternal factors such as exposure to congenital infections or harmful substances, maternal infections (e.g. rubella) or chronic maternal diseases (e.g. diabetes) [12] which can impact intra-uterine growth and post-conception development [10] or maternal nutritional deficiencies. The remaining ~ 60% are of unknown aetiology. Part of the reason so many are of unknown aetiology is that congenital malformations often result from multifactorial causes involving complex interactions between undefined genetic variants and the environment [1, 12]. Given this complexity, the most comprehensive and widely used classification is the system-based approach to diagnosis as per the recently updated International Classification of Diseases 11th Revision (ICD11) [13].

Globally recognized risk factors for congenital malformations include a family history, parental consanguinity, and advanced maternal age [6, 14]. However, context-specific risk factors for congenital malformations among African newborns remain inadequately described, contributing to a gap in the evidence. Key risk factors may include antenatal and perinatal maternal health (such as limited access to antenatal care), maternal nutritional deficiencies (such as in iodine or folate) [15], maternal exposure to infectious agents including syphilis, rubella, Cytomegalovirus, Zika virus, exposure to teratogens (such as pesticides, herbicides, traditional medicines with unknown chemical constituents). Unregulated medicine use, including antibiotics, also poses a potential risk. Other considerations involve exposure to surface water pollution as a source of drinking water or exposure to hazardous waste [16]. The relative contributions of these risk factors may vary by sub-region and season [17].

Robust data on the burden of congenital malformations are lacking in the West Africa sub-region where the neonatal mortality rate is the highest. These data are required to inform the implementation of preventive public health policies aimed at reducing the prevalence of congenital malformations by targeting specific risk factors in the region. In The Gambia, a small country in west Africa, the reported prevalence of congenital malformations was 62.5 per 1000 births in 2006 [1]. A study based on a relatively small cohort indicated that major congenital malformations contributed to 13% of neonatal hospitalisations and up to 8% of neonatal deaths in an urban health facility [18]. This study aims to investigate the clinical epidemiology of easily recognisable congenital malformations in a large cohort of Gambian newborns delivered in health facilities and following a relatively healthy pregnancy. The objectives include determining the prevalence, describing the spectrum of easily recognisable congenital malformations, identifying risk factors associated with the development of malformations, and estimating the impact of malformations on adverse perinatal outcomes, with stratification by severity (major versus minor).

## Methods

### Study design

This analysis is based on data collected as part of the PregnAnZI-2 clinical trial, a phase-III, double-blind, placebo-controlled trial (clinicaltrials.gov NCT03199547) conducted from October 2017 to May 2021 [19]. In this trial, nearly 12,000 pregnant women in The Gambia and Burkina Faso received intrapartum oral azithromycin or placebo, to assess effectiveness on a composite primary outcome of neonatal sepsis or mortality [20]. As the trial intervention was given during labour, it could not have impacted on the aetiology or rates of congenital malformations, hence participants from both trial arms were included in this study. The analysis reported here is restricted to the Gambian cohort of women and their offspring.

### Study setting

The Gambia is the smallest country in West Africa with population of about 2.6 million people in 2020, of whom approximately 570,000 were women of reproductive age, and with a birth rate of 33.7/1000 inhabitants [21]. In 2020, the neonatal mortality rate was 25.7 deaths per 1000 live births [22]. Participants were enrolled at two government-run health facilities located in the Gambia’s urban region; Serekunda Health Centre (SHC), which manages approximately 2,000 deliveries per year; and Bundung Maternal and Child Health Hospital (BMCHH) which serves a larger catchment area of ~ 50,000 inhabitants and manages ~ 5,000 deliveries per year [19]. In both health facilities, antenatal clinics are scheduled monthly; each antenatal clinic includes a health talk, a thorough assessment of pregnant women involving history-taking, physical examinations, and relevant laboratory tests as needed. Additionally, the session includes the administration of tetanus toxoid immunizations and the provision of iron/folate supplementation [23]. At the time of the PregnAnZI-2 trial data collection, SHC lacked the capacity to provide emergency obstetric care, including surgical care; and lacked a neonatal unit. Thus, women in need of an emergency caesarean section were referred to Kanifing General Hospital, 3.4km away from SHC. Neonates born at SHC and requiring hospital care were transferred to the neonatal unit at Kanifing General Hospital or to BMCHH. BMCHH provided emergency obstetric and neonatal care and on rare occasions transferred women or neonates to the national referral centre, Edward Francis Small Teaching Hospital in Banjul (EFSTH), (15.5km away from BMCHH) which has paediatric surgical services and a level 2 + neonatal unit.

The three largest ethnic groups in this Gambian region are Mandinka, Fula and Wolof. Marriage between relatives is common, with 30% of marriages being between first cousins [24]. The climate is typical of that in the Sub-Sahel region, with a short rainy season from June to October and longer dry season from November to May [19].

### Participants, recruitment, and follow-up

The PregnAnZI-2 trial consented pregnant women aged ≥ 16 years during antenatal care at the study health facilities, with enrolment during labour. Exclusion criteria included women with known HIV infection, any chronic or acute conditions, planned caesarean section or known required referral, known severe congenital malformation, intrauterine death or allergy to macrolides, and drugs known to prolong QT interval taken during the last 2 weeks, such as chloroquine, quinine, piperaquine, and erythromycin [19]. In the trial we did not report any participant excluded following a known severe congenital malformation before delivery, likely due to the lack of routine detailed antenatal scans in the Gambia health facilities. This secondary analysis included all women recruited in The Gambia who delivered a live-born newborn at the trial health facilities.

### Study procedures

Written informed consent to participate in the trial was obtained during antenatal visits and verbally confirmed during labour. Women’s socio-demographic and perinatal clinical data were transcribed from the maternal antenatal card and partograph to an electronic case report form (eCRF) by research nurses before discharge from the health facility.

Between 4 and 24 h after birth and before discharge, a detailed clinical examination was carried out by either a trained research clinician or paediatrician, including the newborn’s survival status at the time of assessment (alive or dead), and documentation of any easily recognisable congenital malformation. Newborns who were hospitalised immediately after birth had the same examination in the neonatal ward by the research clinicians. However, for those who died immediately after birth (not seen by research clinicians), the documentation on easily recognisable congenital malformations was based only on the report from the trial nurses present at the time of delivery; this data is not part of this study. Congenital malformations included in this study were those diagnosed by a team of six research clinicians, including five trained medical doctors and one paediatrician. The research clinicians were responsible for assessing all the trial newborns before discharge and diagnosing easily recognizable congenital malformations. Each medical doctor had received formal training in clinical examination and was experienced in assessing newborns. The paediatrician, who had specialized training and experience in paediatric care, provided additional expertise and support. In cases where a medical doctor had doubts regarding the diagnosis of a congenital malformation, they consulted the paediatrician for confirmation. This collaborative approach helped ensure accurate diagnoses and reliable data collection. Both the medical doctors and the paediatrician were tasked with documenting the presence or absence of congenital malformations as part of the study. Detailed investigations such as echocardiography, neuroimaging, abdominal ultrasound, and genetic testing were not routinely available. Congenital malformations requiring urgent surgical intervention were referred to the National Neonatal Referral Hospital (EFSTH). Newborns with congenital malformations needing immediate medical attention were admitted to either the Bundung Maternal and Child Health Hospital (BMCHH) or EFSTH, where they received further management from paediatricians and neonatologists. These referral centres provided ongoing clinical monitoring and appropriate care for affected newborns. Admissions and referrals to the BMCHH or EFSTH neonatal unit were based on either the research clinicians’ or health facility doctors’ assessment of clinical status.

From discharge to the end of the neonatal period (28 days), participants were followed by passive case detection. The study team actively encouraged mothers to attend the health facilities anytime they or their infant were unwell. Active follow-up of both mother and newborn were conducted by study nurses in the form of home visits on day 28 (± 4 days). Newborns requiring further examination by the trial clinicians were referred to the trial sites. The trial clinician or a paediatrician then assessed the mother/newborn and decided whether to admit them for further investigations and management [25]. Written serious adverse events reports were filled for any newborn requiring hospitalisation and management, including those with a congenital malformation. In the event of death, the report included the cause of death based on the trial clinicians’ assessment using clinical notes and results of investigations.

### Outcome and variables of interest

**Fig. 1:**
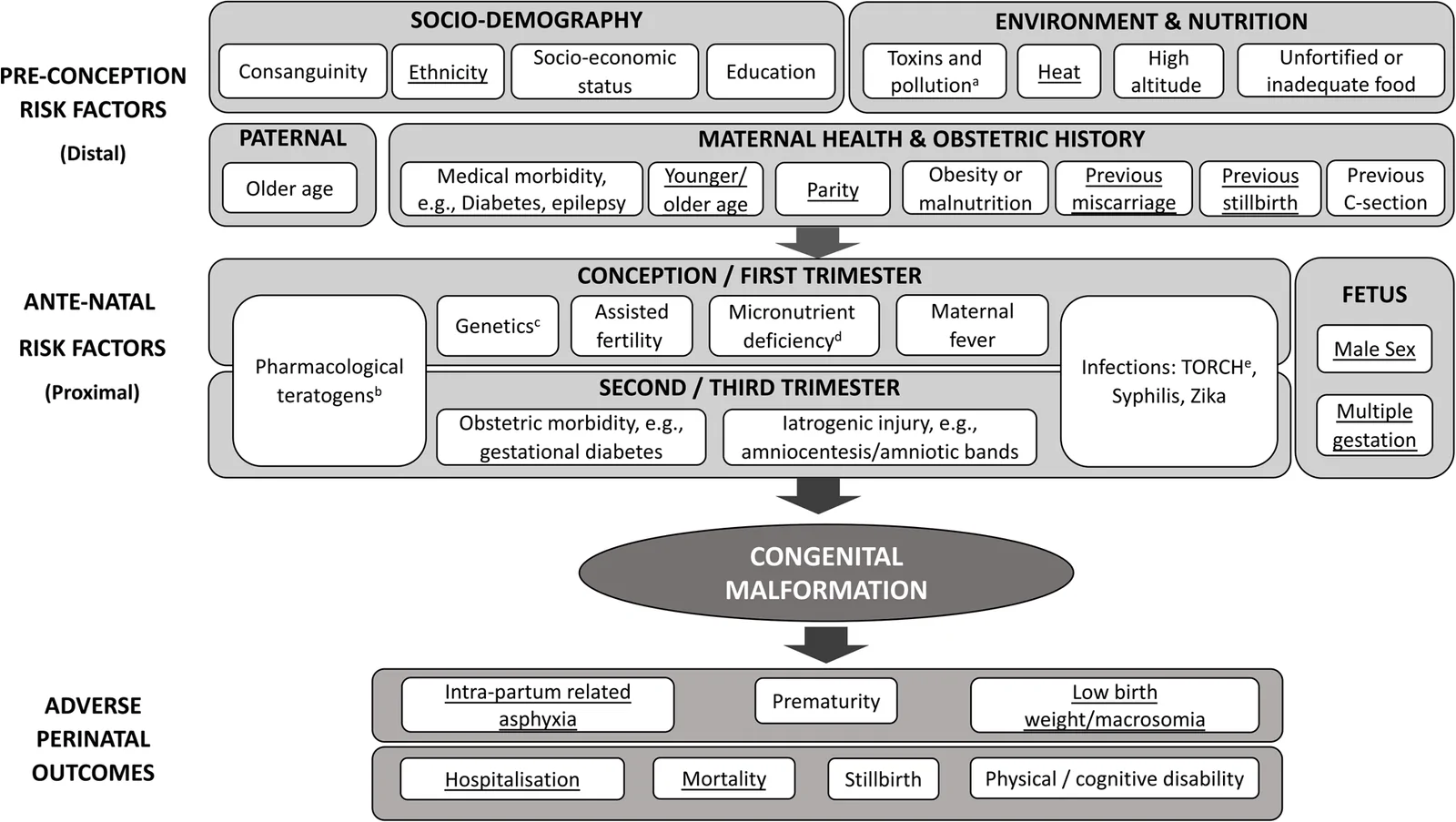
Conceptual Framework to understand risk factors and outcomes of congenital malformations. Underlined = data available in PregnAnZI-2 cohort. **a** Pesticides, lead, mercury, air pollution, passive tobacco smoke; **b** Prescribed medications (e.g., antiepileptics, lithium, opioids), unprescribed medications (e.g., misoprostol), herbal or traditional drugs, alcohol, illicit drugs (e.g., cocaine), tobacco smoking; **c** Genetic mechanisms include single gene mutations, polygenic associations and chromosomal abnormalities; **d** Micronutrient deficiencies include iodine, folic acid, vitamin B12, zinc; **e** TORCH = Toxoplasmosis, rubella, CMV, HSV, HIV

The primary outcome was the occurrence of congenital malformations among live births as detected by the research clinician or paediatrician on clinical examination during the discharge review. Congenital malformations were defined as structural anomalies that were easily recognisable on physical examination. The congenital malformations were categorised and coded by organ-system as per the ICD11 classification [13] by an experienced neonatologist. The severity of congenital malformation was classed as major if it was a structural change with significant medical, social or cosmetic consequences or required medical or surgical intervention, in keeping with WHO guidance [3]. Minor malformations were defined as structural changes posing no significant health problem in the neonatal period and with limited social or cosmetic consequence [3]. Multiple malformations occurring in the same patient were coded separately during descriptive analyses with further classification as major congenital malformation if one or more major congenital malformations was present. To explore the aetiological factors associated with congenital malformation, we developed a conceptual framework based on existing evidence and biological plausibility (Fig. 1). Variables were divided into pre-natal, peri-conception and antenatal exposures, with intersectional relationships described. The conceptual framework also considers potential adverse perinatal outcomes associated with congenital malformations. It guided the choice of variables for the logistic regression analysis (see details below in Statistical analysis).

### Operational definition

The term “Relatively healthy pregnant women” used in our study refers to pregnant women in low-resource health systems such as The Gambia, where antenatal detection of high-risk women/neonates is challenging; this population is representative of pregnant women thought to be at low risk at the time of enrolment. Our study was embedded into a clinical trial that excluded “known” high-risk pregnancies (acute/chronic condition, planned caesarean section, severe congenital malformation, foetal macrosomia, etc.); in practice, often high-risk pregnancies were undiagnosed and were therefore included in the trial due to the lack of rigorous antenatal diagnosis in the trial health facilities.

### Data management and statistical analyses

Research Electronic Data Capture (RedCAP) was used for data capture with consistency checks, data validation and cleaning carried out at regular intervals to ensure high data quality. The data was pseudoanonymised, with each participant assigned a random number. R (version 4.3.1) was used for all analyses. An individual-level analysis was conducted where newborns were classified according to the presence/absence of congenital malformation. Those classified as positive were further classified as positive for a major congenital malformation if one or major congenital malformations was recorded. For analyses describing the distribution of different types of CM, we used the denominator “total number of different occurrences of CM.” In contrast, for analyses involving neonates diagnosed with CM, the denominator “total number of neonates with CM” was used. Crude odds ratios (OR), 95% confidence intervals (CI), and *p*-values were calculated in unadjusted and adjusted analysis to explore associations between congenital malformation and socio-demographic, environmental, and obstetric factors. An adjusted logistic regression model was developed for each independent variable with a *p*-value < 0.2 for any categorical values in the unadjusted regression. Based on the literature and the data available in our dataset, factors considered to be potential confounders (e.g., maternal age) were included in the adjusted analysis. Statistical significance for the adjusted analysis was pre-determined at *p*-value < 0.05.

## Results

### Baseline characteristics

**Fig. 2:**
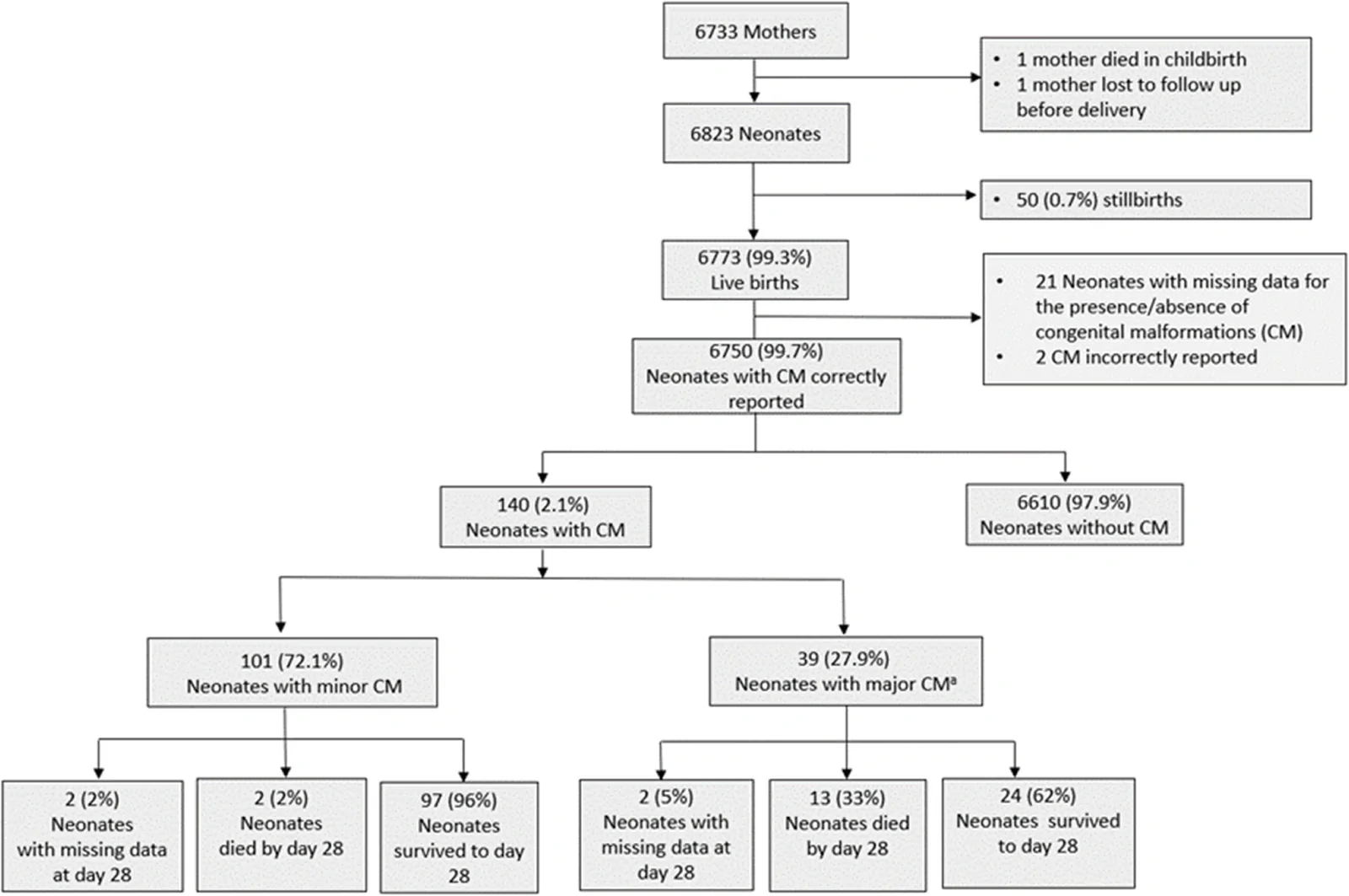
Study Profile for easily recognisable congenital malformations among live-born Gambian neonates following relatively healthy pregnancy. ^a^Newborns with both major and minor malformations were classed as having major malformation

Overall, 6,750 live births were included in the study (Fig. 2), of whom 51.7% (3491/6750) were male, with median birth weight of 3.1kg (interquartile range (IQR) of 2.8–3.4) and predominantly singletons (97.3%, 6571/6750). Out of all live-born neonates, 140 were diagnosed with at least one easily recognizable congenital malformation, resulting in a prevalence of 2.1% (140/6750); among them, 27.9% (39/140) had at least one major easily recognisable congenital malformation (Fig. 2). Multiple malformations were present in 4.3% (6/140) of affected newborns (Table 1).

**Table 1:** Easily recognisable congenital malformations in a cohort of Gambian neonates born following relatively healthy pregnancy, classified and coded as per ICD-11

			**Severity of congenital malformation**	
	**ICD-11 code**	**Total CM**^**a**^ ***N* = 150** **n (%)**	**Minor CM *N* = 106** **n (%)**	**Major CM** ***N* = 44** **n (%)**^**a**^
**Nervous System**		**4 (2.7)**	**0 (0.0)**	**4 (9.1)**
Spina bifida with hydrocephalus^b,c,d^	LA02.00			3
Spina bifida without hydrocephalus	LA02.01			1
**Eyes, Ears, Face, and Neck**		**17 (11.3)**	**14 (13.2)**	**3 (6.8)**
Potters facies^d^	LA5Y		1	
Dysmorphism (unspecified)	LD9Z		1	
Congenital esotropia (squint)	9C80.0		1	
Unspecified abnormality of ear	LA2Z		1	
Skin tag on ear	LA21.Y		1	
Intra-oral mass	LA5Y		1	
Lingual frenulum hypertrophy	LA31Y		3	
Ankyloglossia (tongue tie)	LA31.2		3	
Cleft lip and or palate	LA42			2
Facial cleft	LA51			1
Micrognathia	LA5Y		1	
Anterior neck mass (unspecified)	LA6Z		1	
**Circulatory system**		**6 (4.0)**	**0 (0.0)**	**6 (13.6)**
Congenital heart defect	LA9Z			6
**Diaphragm, abdominal wall or umbilical cord**		**11 (7.3)**	**9 (8.5)**	**2 (4.5)**
Epigastric hernia	LB0Y		1	
Periumbilical hernia	LB0Y		1	
Umbilical hernia	LB03Y		6	
Umbilical abdominal wall defect—unspecified	LB0Z		1	
Omphalocele	LB01			1
Gastroschisis	LB02			1
**Digestive tract**		**3 (2.0)**	**0 (0.0)**	**3 (6.8)**
Oesophageal atresia with tracheo-oesophageal fistula	LB12.10			1
Anal stenosis^b^	LB17			1
Imperforate anus^e^	LB17			1
**Genital system**		**11 (7.3)**	**11 (10.4)**	**0 (0.0)**
Clitoromegaly^d^	LB41.2		2	
Vulval cyst	LB40.1		1	
Extension of the labia minora to the vaginal orifice and anal region	LB40.Y		1	
Vaginal polyp	LB42.Y		1	
Paraphimosis	LB5Y		1	
Penile mass	LB5Z		1	
Micropenis^b^	LB50		1	
Anorchia^b^	LB51		1	
Congenital meatal stenosis^e^	LB5Y		2	
**Breast**		**1 (0.7)**	**1 (0.9)**	**0 (0.0)**
Accessory nipple	LB63		1	
**Skeleton**		**87 (58.0)**	**65 (61.3)**	**22 (50.0)**
Scaphocephaly	LB70.0Y		2	
Spina bifida occulta	LB73		1	
Pectus carinatum	LB73.13		3	
Prominent xyphoid process	LB9Z		1	
Polydactyly	LB78		53	
Syndactyly	LB79		1	
Adactyly of hand^f^	LB99.7			1
Hyperextension of lower limbs	LB93		2	
Congenital deformity of Knee	LB93.Y		1	
Congenital leg defect^f^	LB9Z			2
Talipes equinovalgus	LB98.2Y		1	
Talipes equinovarus^b,c,d,g^	LB98			19
**Skin**		**6 (4.0)**	**6 (5.7)**	**0 (0.0)**
Congenital melanocytic naevus	2F20.2		2	
Congenital dermal melanocytosis	LC10		1	
Port wine stain	LC51		1	
Congenital Ichthyosis	LC7Y		1	
Bilateral pedal lymphoedema	LD0Y		1	
**Chromosomal abnormalities**		**4 (2.7)**	**0 (0.0)**	**4 (9.1)**
Trisomy 21	LD40.0			4

^a^Percentages sum to > 100% because multiple malformations were recorded in 6 out of 140 individuals; ^b^Talipes equinovarus, spina bifida with hydrocephalus, micropenis, anorchia, anal stenosis (*n* = 1); ^c^Talipes equinovarus, spina bifida with hydrocephalus (*n* = 1); ^d^Talipes equinovarus, Potters facies (dysmorphic features of flat nose, recessed chin, epicanthal folds and low set abnormal ears) and clitoromegaly (*n* = 1); ^e^Congenital meatal stenosis + imperforate anus (*n* = 1); ^f^Adactyly of right hand and congenital lower limb malformations (*n* = 1); ^g^talipes equinovarus and other unspecified lower limb malformations (*n* = 1)

### Types of easily recognisable congenital malformations

**Fig. 3:**
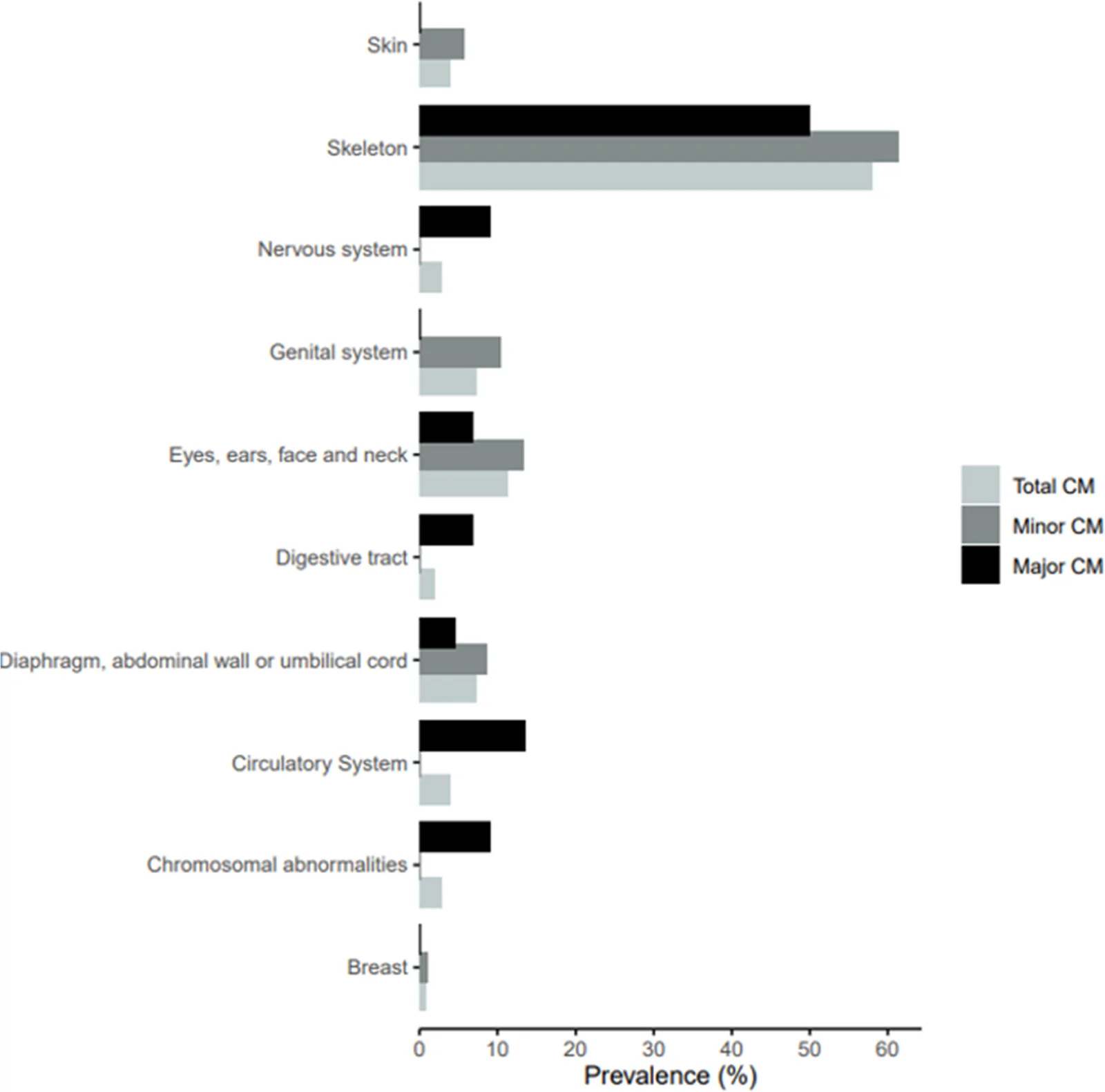
Prevalence of easily recognisable congenital malformations among Gambian neonates by type and severity of the malformation classified as per ICD-11. Total CM: prevalence of each class of congenital malformation among the total number of congenital malformations recorded in the study (*N* = 150). Minor CM: prevalence of minor congenital malformation among each class congenital malformation among the total number of minor congenital malformations recorded in the study (*N* = 106). Major CM: prevalence of major congenital malformation among each class of congenital malformation among the total number of major congenital malformations recorded in the study (*N* = 44)

In total, 150 different occurrences of easily recognisable congenital malformations were recorded. Nearly one-third (29.3%, 44/150) of them were classified as major (Fig. 2, Table 1), with the musculoskeletal system comprising the largest proportion of easily recognisable congenital malformations (58.0%, 87/150) (Table 1), (Fig. 3). Talipes equinovarus was the most frequently observed major malformation (43%, 19/44) followed by suspected congenital heart defects (13.6%, 6/44) and spina bifida with or without congenital hydrocephalus (9.1%, 4/44) (Table 1). There were 38 distinct minor malformations, with polydactyly the most common (50.0%, 53/106) (Table 1), (Fig. 3).

### Risk factors for easily recognisable congenital malformations

A history of previous miscarriage was associated with increased odds of the newborn having a congenital malformation (OR:1.70, 95%CI:1.00–2.77, p-value = 0.03). No other risk factors were significantly associated with malformations (Table 2).

**Table 2:** Maternal, prenatal, and antenatal factors associated with easily recognisable congenital malformations in a cohort of Gambian neonates born following relatively healthy pregnancy

		**Unadjusted**			**Adjusted**	
	N	**n (%)** ***n* = 140**	**OR (95%CI)**	***p*-value**	**OR (95%CI)**	***p*-value**
**Maternal factors** *(n* = *6750)*						
*Age (Years)*				0.30		0.31
Median (IQR)	27 (23–31)					
20–35	5453 (80.8)	106 (1.9)	1		1	
16–19	632 (9.4)	17 (2.7)	1.39 (0.80–2.27)		1.24 (0.71–2.04)	
> 35	665 (9.8)	17 (2.6)	1.32 (0.76–2.16)		1.46 (0.84–2.39)	
*Mother’s Ethnicity*				0.45		
Mandinka	2701 (40.0)	57 (2.1)	1			
Fula	1212 (18.0)	28 (2.3)	1.10 (0.69–1.72)			
Wolof	1040 (15.4)	17 (1.6)	0.77 (0.43–1.30)			
Jola	870 (12.9)	23 (2.6)	1.26 (0.76–2.03)			
Others	927 (13.7)	15 (1.6)	0.76 (0.41–1.32)			
*Father’s Ethnicity*^*a*^				0.36		
Mandinka	2611 (38.7)	60 (2.3)	1			
Fula	1158 (17.1)	23 (2.0)	0.86 (0.52–1.38)			
Wolof	1165 (17.3)	23 (2.0)	0.86 (0.52–1.37)			
Jola	830 (12.3)	21 (2.5)	1.10 (0.65–1.80)			
Others	985 (14.6)	13 (1.3)	0.57 (0.30–1.01)			
*Parity*				0.70		
Para > = 4	2513 (37.3)	55 (2.2)	1.05 (0.67–1.68)			
Primiparous	1680 (24.9)	37 (2.2)	1.06 (0.65–1.74)			
Para 2	1393 (20.6)	29 (2.1)	1			
Para 3	1164 (17.2)	19 (1.6)	0.78 (0.43–1.39)			
*Season of delivery*				0.34		
Dry	4568 (67.7)	100 (2.2)	1			
Rainy	2182 (32.3)	40 (1.8)	0.83 (0.56–1.22)			
*Previous stillbirths*^*b*^				0.66		
No	6636 (98.3)	137 (2.1)	1			
Yes	113 (1.7)	3 (2.7)	1.29 (0.26–3.96)			
***Previous miscarriage***^***c***^				**0.03**		**0.04**
No	6138 (91.0)	120 (2.0)	1		1	
Yes	610 (9.0)	20 (3.3)	1.70 (1.00–2.77)		1.72 (1.02–2.37)	
*Previous intake of traditional medicine*^*d*^				0.88		
No	5573 (82.6)	115 (2.1)	1			
Yes	1173 (17.4)	25 (2.1)	1.03 (0.64–1.61)			
**Neonatal factors** *(n* = *6750)*						
*Sex*				0.34		
Female	3259 (48.3)	62 (1.9)	1			
Male	3491 (51.7)	78 (2.2)	1.18 (0.83–1.68)			
*Plurality of birth*				-		
Singleton	6571 (97.3)	140 (2.1)				
Twin	179 (2.7)	**0 (0.0)**	-			

^a^Ethnicity father missing in *n* = 1

^b^History of stillbirths missing in *n* = 1

^c^History of miscarriage missing in *n* = 2

^d^ Previous intake of traditional medicine missing in *n* = 4

### Adverse perinatal outcomes

**Fig. 4:**
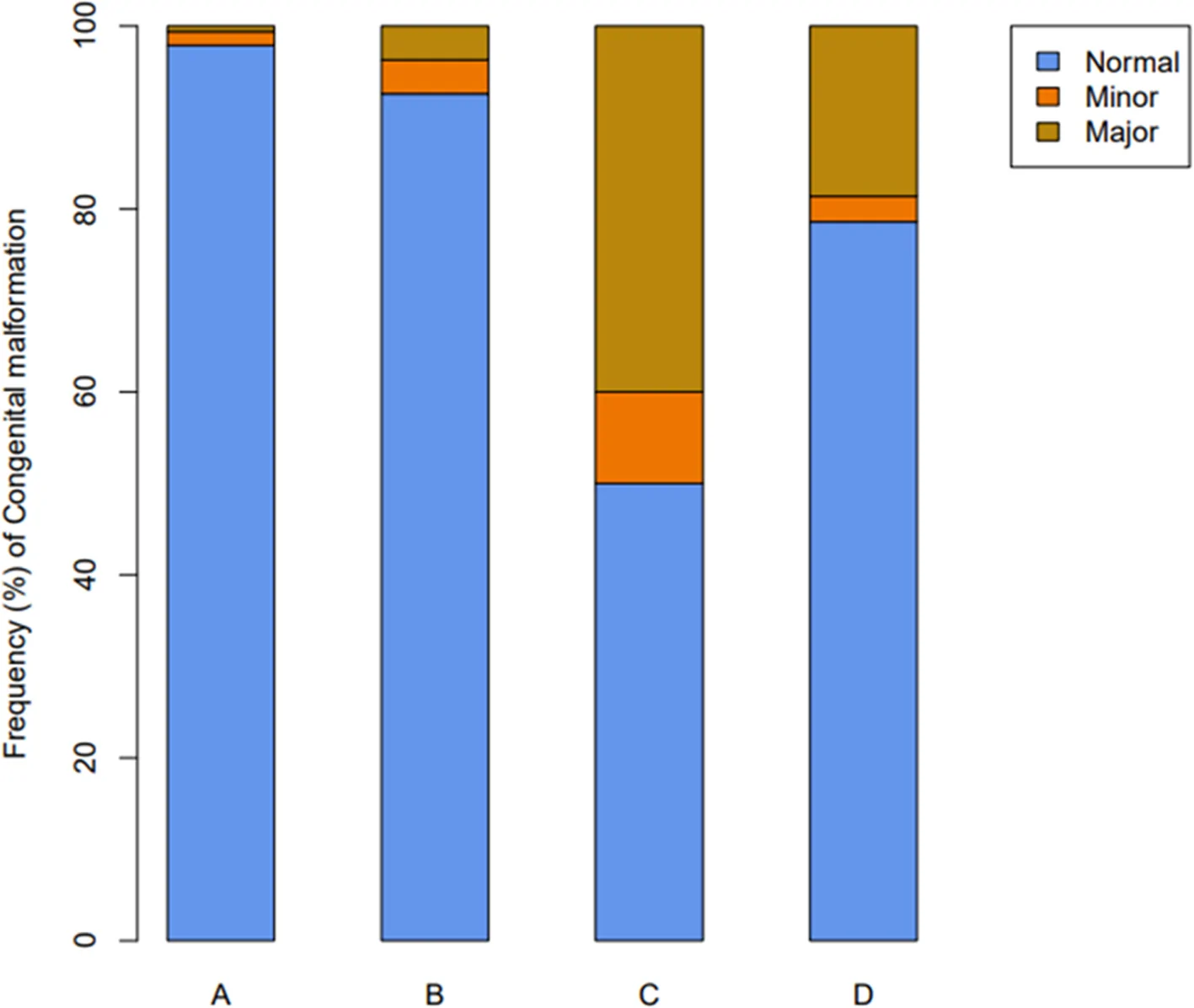
Proportion of neonates with easily recognisable congenital malformations among births, admissions, deaths within 24 h and deaths by day 28 in a cohort of Gambian neonates born following relatively healthy pregnancy. **A** Proportion of neonates with congenital malformation among births. **B** Proportion of neonates with congenital malformation among admissions (0–28 days). **C** Proportion of neonates with congenital malformation among neonatal deaths within 24 h after birth). **D** Proportion of neonates with congenital malformation among overall neonatal deaths (0–28 days)

**Fig. 5:**
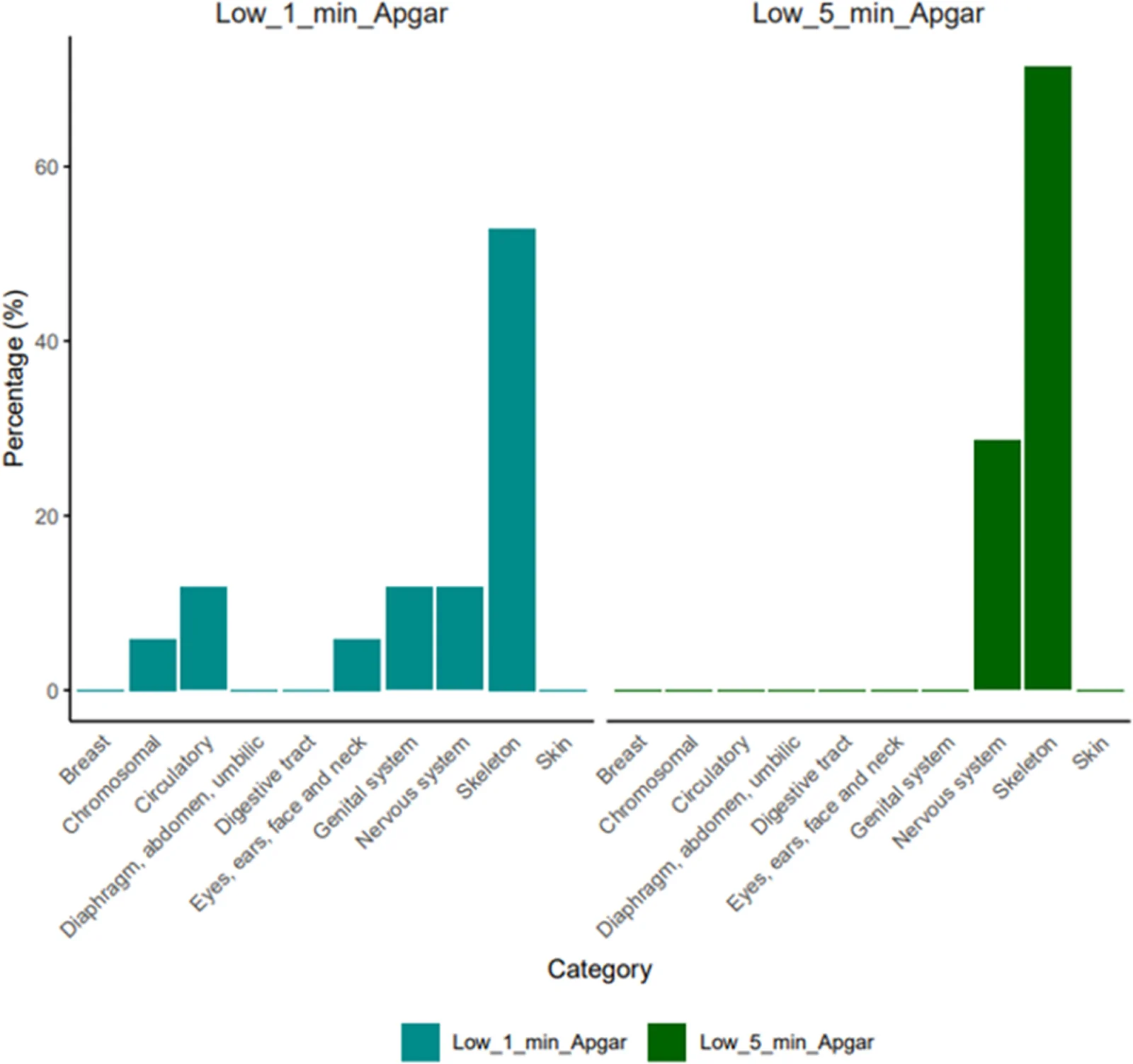
Proportion of each easily recognisable congenital malformations among neonates with low Apgar scores. Low_1_min_Apgar score, *N* = 14. Low_5_min_Apgar score, *N* = 6

**Fig. 6:**
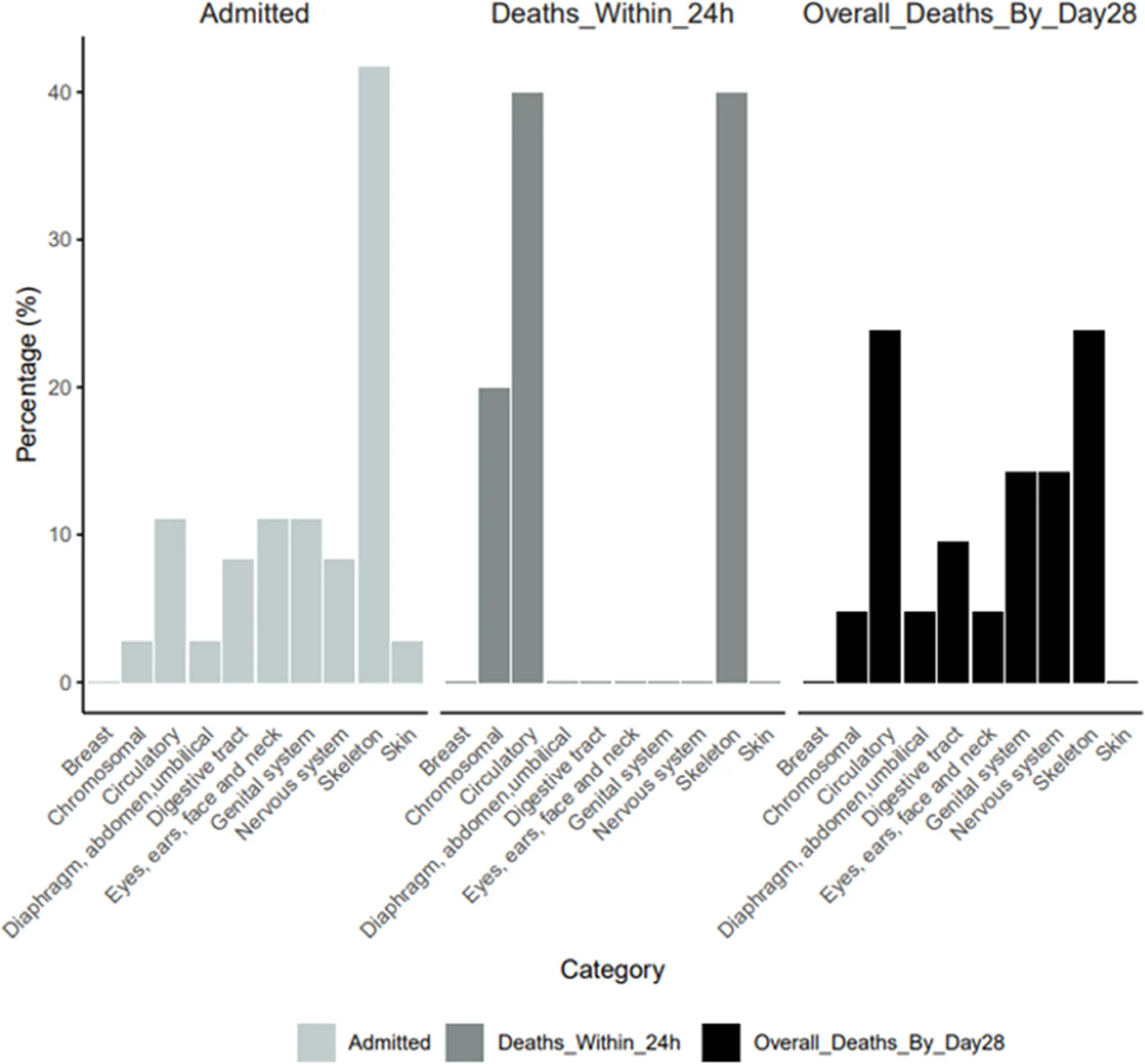
Proportion of each type of easily recognisable congenital malformations among, admissions, deaths within 24 h, and deaths by day 28 after birth in a cohort of Gambian neonates. Admitted, *N* = 28. Deaths_within_24h, *N* = 5. Overall_Deaths_By_Day28, *N* = 15

Newborns with an easily recognisable congenital malformation were more likely to have an adverse perinatal outcome compared to those without (Fig. 3, Table 3). Higher odds of both low 1-min Apgar score (OR:3.09, 95%CI:1.62–5.48, *p*-value < 0.001) and low 5-min Apgar score (OR:6.10, 95%CI:2.10–14.62, *p*-value < 0.001) were observed. The odds of being admitted to hospital were more than four-fold higher than in newborns with no malformation (OR:4.42, 95%CI:2.77–6.88, *p*-value < 0.001); all mortality outcomes were higher, especially death within 24 h of delivery (OR:48.75, 95%CI:11.08–215.62, *p*-value < 0.001) (Table 3, Fig. 3). Irrespectively of the severity of congenital malformations (minor or major), hospitalisation during the neonatal period was higher among newborns with congenital malformations than in those without (Table 4). In total, 15 out of 70 neonatal deaths (21.4%) occurred in newborns with congenital malformations and 50% (5 out of 10) of deaths within the first 24 h after birth were among newborns with congenital malformations (Table 4); these early and overall neonatal deaths were more common among those with major congenital malformations (Fig. 4). Among neonates with congenital malformations, those affecting the skeleton were most frequently recorded among admitted neonates. Similarly, skeletal, circulatory system and chromosomal congenital malformations were more commonly associated with deaths by day 28 after birth. Figures 5 and 6 provide details on perinatal outcomes related to easily recognizable congenital malformations.

**Table 3:** Adverse perinatal outcomes associated with easily recognisable congenital malformations in a cohort of Gambian neonates born following relatively healthy pregnancy

	**Congenital malformation,**		**OR (95% CI)**	***p*-value**
	Yes (*n* = 140) n (%)	No (*n* = 6610) n (%)		
Birth weight^a^				
Normal	129 (2.1)	5949 (97.9)	1.04 (0.56–2.15)	0.90
Low	11 (2.0)	527 (98.0)		
Macrosomia	**0 (0.0)**	132 (100.0)	-	
1-min Apgar score^b^				** < 0.001**
Normal	126 (1.9)	6366 (98.1)	1	
Low	14 (5.8)	229 (94.2)	3.09 (1.62–5.48)	
5-min Apgar score^c^				** < 0.001**
Normal	134 (2.0)	6547 (98.0)	1	
Low	6 (11.1)	48 (88.9)	6.10 (2.10–14.62)	
Admission				** < 0.001**
No	112 (1.8)	6256 (98.2)	1	
Yes	28 (7.3)	354 (92.7)	4.42 (2.77–6.83)	
Death within 24 h after delivery				** < 0.001**
No	135 (2.0)	6605 (98.0)	1	
Yes	5 (50.0)	5 (50.0)	48.75 (11.08–215.62)	
Death between 24 h and 28days				** < 0.001**
No	121 (1.9)	6301 (98.1)	1	
Yes	10 (16.7)	50 (83.3)	10.40 (4.60–21.39)	
Overall death on day 28				** < 0.001**
No	121 (1.9)	6301 (98.1)	1	
Yes	15 (21.4)	55 (78.6)	14.18 (7.23–26.33)	

^a^birth weight missing in *n* = 2

^b^1-min Apgar score missing in *n* = 15

^c^5-min Apgar score missing in *n* = 15

^d^Survival status on day 28 missing in *n* = 258

**Table 4:** Adverse perinatal outcomes associated with easily recognisable congenital malformations stratified by degree of congenital malformation in a cohort of Gambian neonates born following relatively healthy pregnancy

	**Major** **(*n* = 39)**	**Normal** **(*n* = 6610)**	**OR (95%CI)**	***p*-value**	**Minor** **(*n* = 101)**	**Normal** **(*n* = 6610)**	**OR (95%)**	***p*-value**
*Birth weight*^*a*^								
Normal	34(0.6)	5949 (99.4)	1.65 (0.50–4.29)	0.29	95 (1.6)	5949 (98.4)	1.40 (0.62–3.94)	0.42
Low	5 (0.9)	527(99.1)			6 (1.1)	527 (98.9)		
Macrosomia	**0 (0.0)**	132 (100.0)	-		**0 (0.0)**	132 (100.0)	-	
*1-min Apgar*^*b*^* score*								
Normal	30 (0.5)	6367 (99.5)			96 (1.5)	6366 (98.5)		
Low	9 (3.8)	230 (96.2)	8.33 (3.43–18.27)	** < 0.001**	5 (2.1)	229 (97.9)	1.45 (0.45–3.54)	0.42
*5-min Apgar*^*c*^ *score*								
Normal	36 (0.5)	6547 (99.5)			98 (1.5)	6547 (98.5)		0.04
Low	**3 (5.9)**	48 (94.1)	11.35 (2.16–37.88)	** < 0.001**	**3 (5.9)**	48 (94.1)	4.17 (0.82–13.30)	
*Neonatal Admission*								
No	25 (0.4)	6256 (99.6)			87 (1.4)	6256 (98.6)		
Yes	14 (3.8)	354 (96.2)	9.89 (4.70–19.97)	** < 0.001**	14 (3.8)	354 (96.2)	2.84 (1.48–5.09)	** < 0.001**
*Death within 24h*								
No	35 (0.5)	6605 (99.5)			100 (1.5)	6605 (98.5)		
Yes	**4 (44.4)**	5 (55.6)	148.91 (28.37–728.18)	** < 0.001**	**1 (16.7)**	5 (83.3)	13.19 (0.28–119.05)	0.09
*Death between 24h* and 28 days								
No	24 (0.4)	6301 (99.6)			97 (1.5)	6301 (98.5)		
Yes	9 (15.3)	50 (84.7)	47.06 (18.30–111.63)	** < 0.001**	**1 (2.0)**	50 (98.0)	1.30 (0.03–7.75)	0.80
*Overall death on day* 28^d^								
No	24 (0.4)	6301 (99.6)			97 (1.5)	6301 (98.5)		
Yes	13 (19.1)	55 (80.9)	61.81 (27.40–133.95)	** < 0.001**	**2 (3.5)**	55 (96.5)	2.36 (0.28–9.18)	0.22

^a^ birth weight missing in *n* = 2

^b^ 1-min Apgar score missing in *n* = 15

^c^ 5-min Apgar score missing in *n* = 15

^d^ Survival status on day 28 missing in *n* = 258

## Discussion

This large cohort study of “relatively healthy pregnant women” in The Gambia found that 2.1% of newborns had an easily recognisable congenital malformation. Despite this seemingly low prevalence, newborns with malformations constituted more than one in five of all neonatal deaths. Additionally, the presence of these easily recognisable congenital malformations was strongly associated with other adverse perinatal outcomes such as low Apgar scores and hospitalisation, even mild congenital malformations were strongly associated with neonatal hospitalisation. Musculoskeletal congenital malformations such as talipes equinovarus (most common major) and polydactyly (most common minor) were most frequently observed. History of miscarriage was the only identified risk factor for easily recognisable congenital malformations in our cohort.

The observed prevalence of 2.1% of congenital malformations aligns with findings from other West African studies despite notable variations in prevalence across different countries in Sub-Saharan Africa [26] and differences in the methods to define these malformations [27]. Notably, within a single country such as Nigeria, prevalence has been reported to range widely, from 1.3% to 6.3% [28]. A subgroup analysis for a metanalysis showed a prevalence of 0.7% for congenital malformations in West Africa [29]. In our study, easily recognisable congenital malformations most frequently affected the musculoskeletal system, with polydactyly having the highest frequency, followed by talipes equinovarus. Similar observations were made in other resource-limited settings and hospital based studies in Southwestern Nigeria [30], and Uganda [31], where the musculoskeletal system accounted for 43.8%, and 45.7% of malformations, respectively. In all these studies, including ours, the diagnosis of congenital malformations relied on clinical examination. This approach may lead to an over-estimation of the prevalence of musculoskeletal malformations as they are more easily identifiable in the absence of radiological investigations.

Despite the relative low prevalence of easily identifiable congenital malformations among the study newborns, 1 in 2 neonatal deaths within the first 24 h of life occurred among newborns with congenital malformations, and this extended to 1 in 5 newborns who died within the neonatal period. Our findings, indicating an increased risk of mortality among newborns with easily recognisable congenital malformations, align with a prior, smaller study conducted in the same Gambian region involving a similar population. The earlier study reported 36-fold increased odds of mortality for neonates with major malformation [18]. Such strong associations with mortality have also been found in other African studies conducted in Eritrea and Tanzania (32, 33). However, in those studies, congenital malformations were present in 10.1% and 9.0% of neonatal deaths, respectively, compared to our higher figure of 21.4%. These disparities may stem from differences in the study design. The study in Eritrea was a hospital-based retrospective cross-sectional study and focused on admitted newborns, potentially missing deaths among those newborns who died very soon after delivery or were delivered in other health facilities. The study in Tanzania, being registry-based, included exclusively newborns from the neonatal care unit, possibly overlooking early deaths. In the same line, the association of congenital malformation with other poor outcomes such as low-1 min and −5 min Apgar scores, hospitalisation to neonatal intensive care unit, and higher risk of death within first 24 h after delivery suggests that for these newborns intra-uterine hypoxia and neonatal encephalopathy may be involved in the causal pathway to mortality (see Fig. 1). Congenital malformations, especially those of the hip, central nervous system, musculoskeletal system, are associated with breech presentation, [34] which is strongly linked to an increased risk of hypoxic-anoxic events [35] and prolonged labour with potential progression to intra-partum related asphyxia [36] and subsequent neonatal encephalopathy [37]. A study conducted in The Netherlands also showed a significant association between a wide variety of congenital malformations and newborn encephalopathy, with the congenital malformation considered to be the probable cause of the encephalopathy in 36% of cases [38]. The strong association between congenital malformations and adverse perinatal outcomes observed in our study highlights the importance of antenatal detection of congenital malformations to enable appropriate and timely health facility delivery to avoid intrapartum-related asphyxia, neonatal encephalopathy, and potentially avoidable death. Routine antenatal surveillance using detailed second trimester ultrasound, could improve outcomes by informing delivery planning and timely specialist referrals for these high-risk newborns [39, 40].

A history of miscarriage was associated with congenital malformations in our study. This factor was the only significant risk factor identified in the unadjusted analysis. Similar findings have been reported in studies conducted in low- and middle-income countries. In India, 16.5% of cases of congenital malformations had a history of previous miscarriage [41]. Likewise, a study across 72 hospitals in South America revealed an increased risk of congenital malformation in mothers with previous fetal loss [42].. Even though the causal pathway for the association between congenital malformations and previous miscarriage is not fully understood, it has been postulated that spontaneous miscarriage is often correlated with genetic abnormalities incompatible with life [43]. Other explanations include recurrent maternal exposures, ranging from nutritional deficiencies (e.g., folic acid), teratogens or infections (e.g. syphilis) that may result in either in-utero demise or disruption of embryogenesis. Therefore, a history of miscarriage could serve as a red flag for potential higher risk of congenital malformation and provide an opportunity for enhanced antenatal care and detailed ultrasound scans for women experiencing recurrent miscarriages. Earlier in-utero detection of congenital malformations would enable family-centered counselling, delivery at an appropriate health facility and prompt postnatal investigation and management, all of which have potential benefits for newborns and their families.

The limitation of our study includes limited access to radiological investigations, which prevented extensive and precise diagnosis of congenital malformations, likely under-estimating the total prevalence, especially for multiple malformations and congenital heart defects; However, our study specifically targeted easily recognisable congenital malformations typically identifiable through clinical examination, such as limb deformities, cleft lip/palate, and neural tube defects. Given this targeted approach, we believe that the potential for detection bias is minimised. However, we acknowledge that we cannot exclude the possibility that some subtle congenital malformations were missed due to human error. For generalisability, future studies should target broader populations and use standardized clinical assessment protocols, including radiological investigations. The inclusion of only relatively healthy pregnant women and their live-born offspring has probably underestimated the prevalence of malformations and further population-based research including both women with complicated pregnancies and stillbirths is needed. However, considering our contextual definition of “relatively healthy women”, we believe our findings contribute to the regional and global literature on this important topic and highlight the important contribution of congenital malformations towards neonatal hospitalisations and mortality. As this was a post-hoc study using an existing dataset, comprehensive data regarding other known risk factors for congenital malformations were not available, e.g., use of medications and nutritional intake during pregnancy or consanguinity between parents. Despite this data limitation, the study's key findings are scientifically solid, and represent clinical data that is easily ascertained in practice in our setting. The inclusion of only two public health facilities from The Gambia, both situated in urban areas, limits generalisability of our results to the entire country and other rural regions of West Africa. More research is needed to determine prevalence and risk factors in rural areas as differences may exist in nutritional, environmental and genetic exposures.

## Conclusion

Even among relatively healthy women easily recognisable congenital malformations are associated with substantial neonatal morbidity and mortality and are an important public health issue. Improved antenatal diagnosis of congenital malformations is urgently required to optimise delivery strategies and improve perinatal outcomes, especially for women with history of previous miscarriage. A nationwide database/register for congenital malformations in the Gambia is also essential for ongoing surveillance/monitoring and to inform and guide policy. Raising awareness about the importance of regular antenatal visits and early detection of congenital malformations among pregnant women can encourage better use of diagnostic services. Additionally, focused training for healthcare providers on recognizing congenital malformations, expanding access to ultrasound screening, and strengthening referral systems to advanced diagnostic centres are key steps toward improving the detection and care of congenital malformations in The Gambia.

## Data Availability

Data may be obtained from a third party and are not publicly available. The clinical data has been collected following provision of informed consent under the prerequisite of strict participant confidentiality. Qualified researchers may request access with the Gambia Government/MRC Joint Ethics Committee. The review process and release of data will be facilitated by MRC Unit The Gambia (http://www.mrc.gm/) through the Head of Governance at MRCG. Access will not be unduly restricted.

## Abbreviations

DALY: Disability Adjusted Life Years
SSA: Sub-Saharan Africa
SDG: Sustainable Development Goal
SHC: Serekunda Health Centre
BMCHH: Bundung Maternal and Child Health Hospital
eCRF: Electronic Case Report Form
